# Nafcillin and Hypokalemia: A Forgotten Adverse Effect

**DOI:** 10.7759/cureus.92012

**Published:** 2025-09-10

**Authors:** Tambi Isaac, Samy Matta, Mahfuza Khan, Konstantinos Mouskas, Roxana Lazarescu

**Affiliations:** 1 Medical Academy, Kabardino-Balkarian State University, Nalchik, RUS; 2 Internal Medicine, Wyckoff Heights Medical Center, New York, USA; 3 Internal Medicine, Alexandria University, Alexandria, EGY; 4 Surgery, Wyckoff Heights Medical Center, New York, USA; 5 Internal Medicine, Touro College of Osteopathic Medicine, New York, USA

**Keywords:** drug-induced electrolyte disturbance, fractional excretion of potassium, hypokalemia, nafcillin, potassium depletion, renal potassium wasting

## Abstract

Hypokalemia is a common yet often overlooked electrolyte disturbance in hospitalized patients. Among the various etiologies, drug-induced hypokalemia remains underrecognized. Nafcillin, a beta-lactam antibiotic widely used for methicillin-sensitive *Staphylococcus aureus* (MSSA) infections, has been infrequently reported as a cause of renal potassium wasting.

We present the case of a 67-year-old male hospitalized for MSSA bacteremia complicated by epidural and shoulder abscesses, ultimately requiring extensive surgical interventions and long-term intravenous nafcillin therapy. During his hospital course, the patient developed persistent hypokalemia that was resistant to supplementation. A thorough evaluation excluded gastrointestinal, renal, and endocrine causes. The calculated fractional excretion of potassium (FEK) was 17.7%, indicating renal potassium wasting. The hypokalemia was attributed to nafcillin therapy and managed successfully with aggressive potassium and magnesium repletion.

This case highlights nafcillin as a potential but underreported cause of renal potassium wasting. Clinicians should consider nafcillin-induced hypokalemia in patients receiving prolonged therapy, particularly when hypokalemia persists despite adequate supplementation and resolution of other contributing factors. Routine electrolyte monitoring and early intervention can mitigate complications and ensure safe continuation of antibiotic treatment.

## Introduction

Hypokalemia, defined as a serum potassium level below 3.5 mmol/L, is a common electrolyte disturbance frequently encountered in hospitalized patients. Its etiology is diverse, but drug-induced hypokalemia is a significant and often overlooked contributor. Certain medications - including diuretics, corticosteroids, and antibiotics - can promote potassium loss through renal excretion, intracellular shifts, or gastrointestinal mechanisms [1, 2].

Medication-induced hypokalemia is among the most frequently observed electrolyte abnormalities in the inpatient setting and is associated with prolonged hospitalizations, increased morbidity, and mortality, especially with K^+^ < 2.9 mmol/L [3].

Nafcillin is a narrow-spectrum, second-generation beta-lactam antibiotic used primarily for treating infections caused by methicillin-sensitive Staphylococcus aureus (MSSA). Despite its widespread use, reports of nafcillin-associated hypokalemia are limited [4, 5].

This case report aims to emphasize the importance of early recognition of nafcillin-induced hypokalemia. By bringing attention to this potential side effect, we hope to promote clinician awareness and stimulate the development of monitoring guidelines and prophylactic supplementation protocols during nafcillin therapy.

## Case presentation

Our patient is a 67-year-old male with a medical history of hypertension, multiple falls, benign prostatic hyperplasia, and alcohol use disorder. He was presented to the emergency department after being found on the floor by his landlord. The patient reported generalized weakness, poor oral intake, and multiple episodes of non-bilious, non-bloody vomiting. He denied fever, chest pain, syncope, palpitations, dyspnea, dizziness, dysuria, or bowel changes. Initial vitals included a blood pressure of 124/69 mmHg, heart rate of 104 bpm, temperature of 97.5 °F, and oxygen saturation of 100% on room air.

On examination, the patient appeared frail and dehydrated. He had abrasions on both knees consistent with recent falls. Pupils were equal and reactive to light. Cardiac and pulmonary exams were unremarkable. Pitting edema was noted in the right lower extremity. Peripheral pulses were palpable (+2), and neurologically, the patient exhibited 4/5 strength with intact sensation.

**Figure 1:**
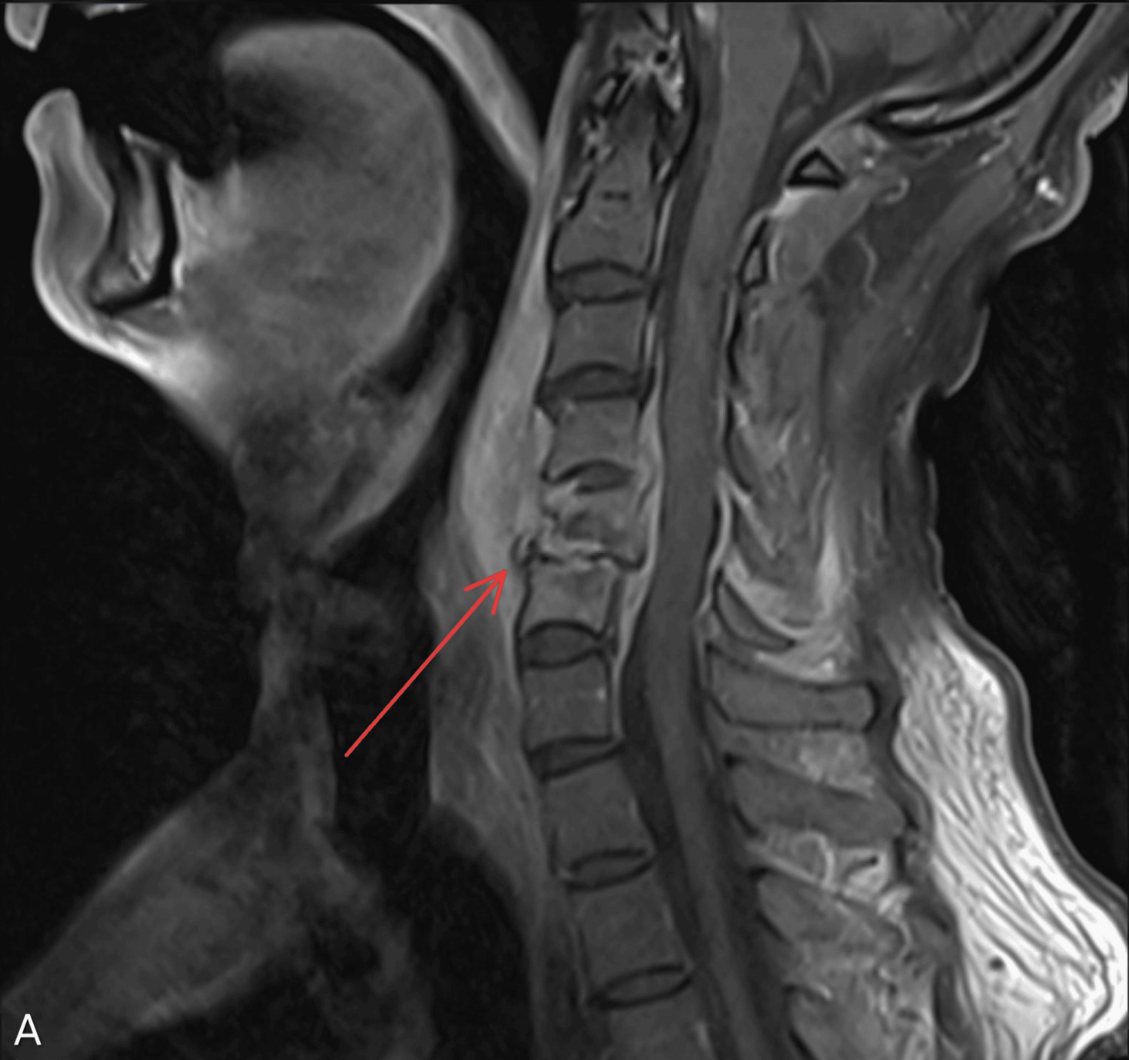
MRI of the cervical spine with the red arrow showing osteomyelitis at C5-C6 with evolving infectious process in the adjacent tissue

**Figure 2:**
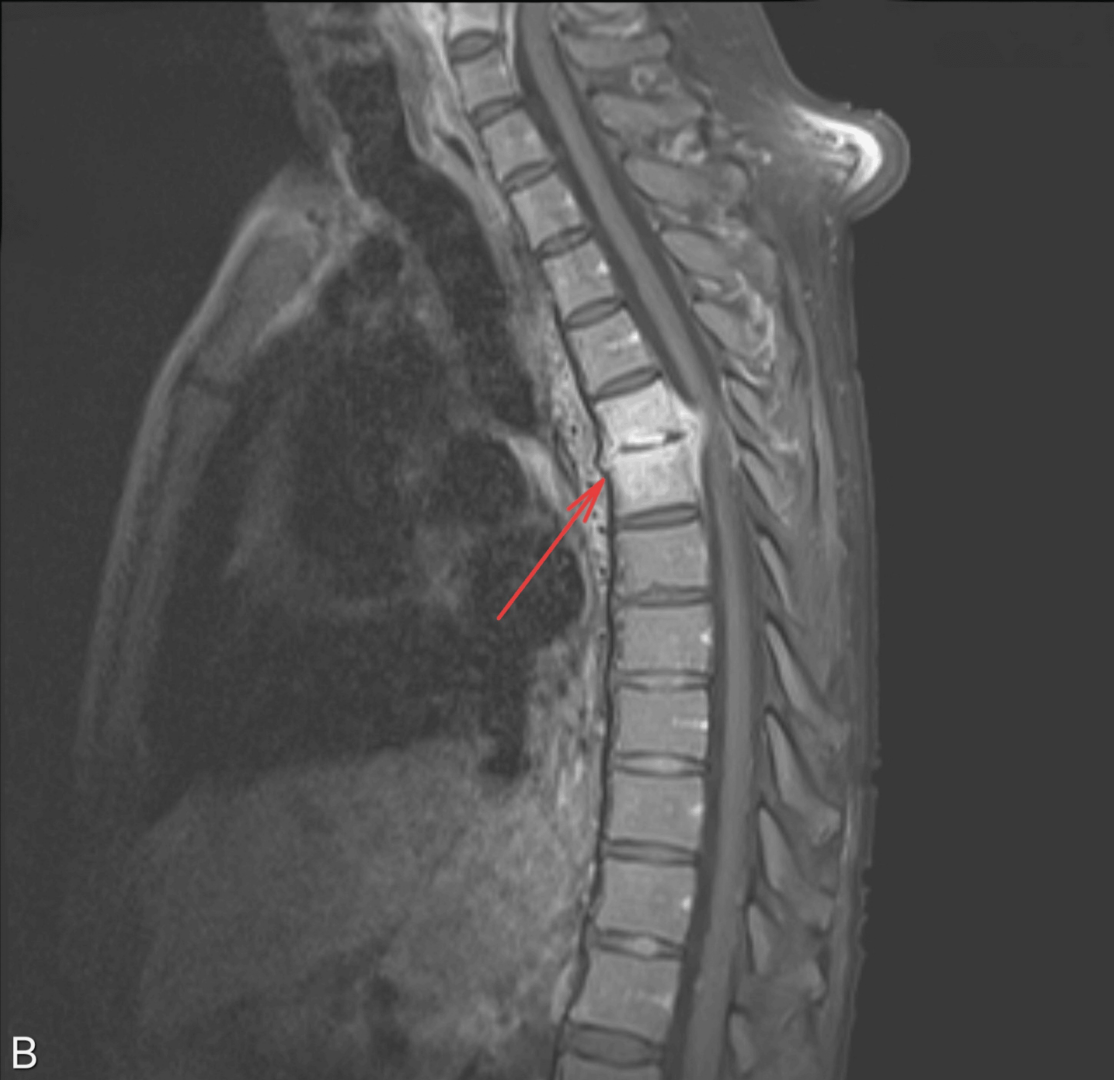
MRI of the thoracic spine with the red arrow showing osteomyelitis and evolving abscess at the level T5-T6

**Figure 3:**
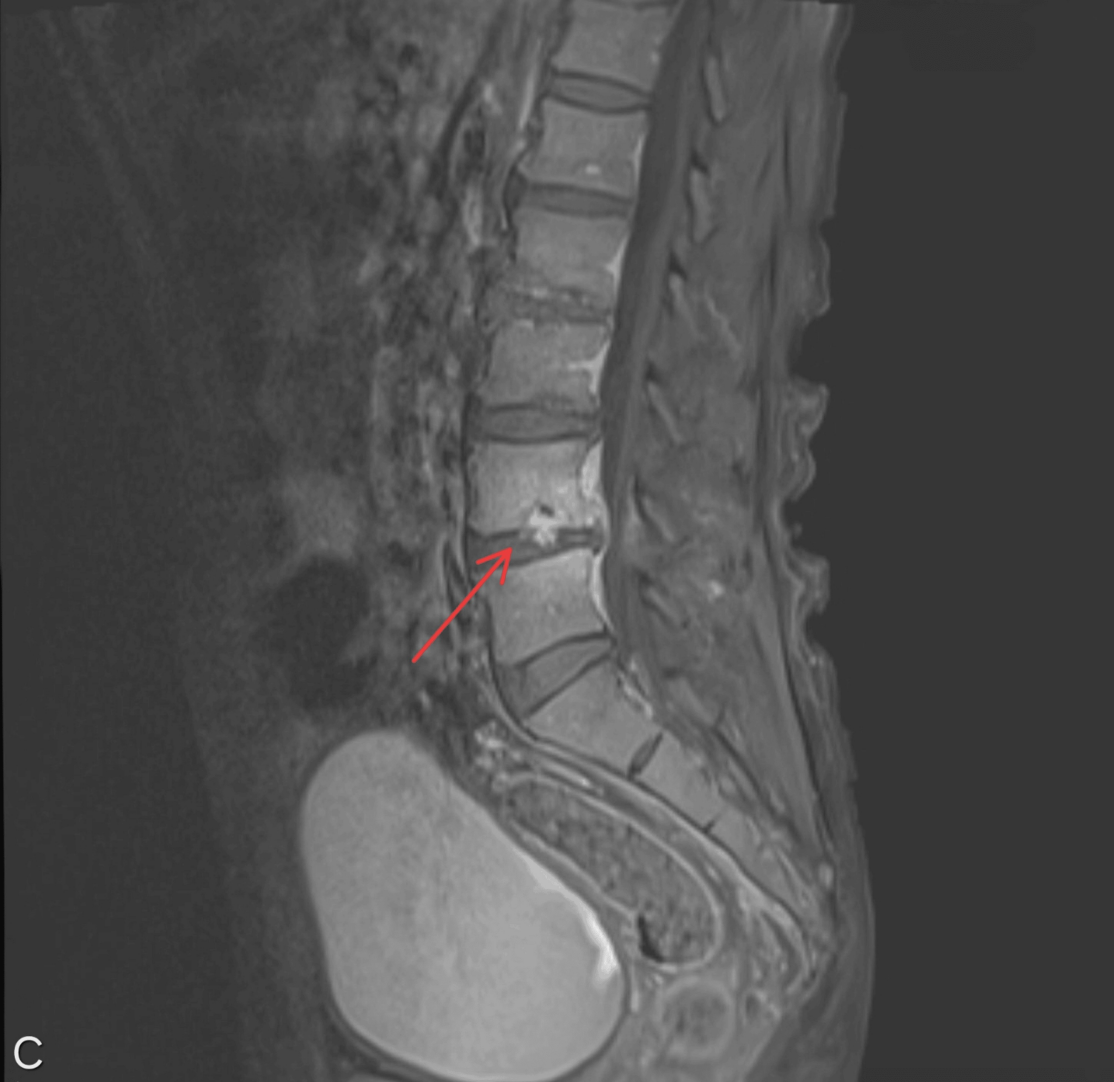
MRI of the lumbar spine with the red arrow showing evolving osteomyelitis at the level L4-L5

Laboratory tests revealed markedly elevated creatine phosphokinase (CPK) at 3,169 U/L (26-308.0 U/L), consistent with rhabdomyolysis. The white blood cell count was 19.00 K/μl (4.5-10.9 K/μl), C-reactive protein (CRP) was 129 mg/L (0.20-3.00 mg/L), and erythrocyte sedimentation rate (ESR) was 106 mm/hr (0-20 mm/hr). Blood cultures were positive for *Methicillin-sensitive Staphylococcus aureus *(MSSA). MRI of the thoracic and cervical spine revealed osteomyelitis with progressing epidural abscesses at C5-C6 and T5-T6 (Figures 1, 2), canal stenosis and cord compression. Also, an evolving abscess at the L4-L5 (Figure 3). CT chest demonstrated right shoulder fluid collections.

The patient was admitted for rhabdomyolysis and was started on intravenous Lactate Ringer's solution at a rate of 100 mL/hour, along with doxycycline 100 mg intravenously every 12 hours.

Orthopedic service was consulted, given the CT finding as mentioned above, to rule out septic arthritis. The patient was taken to the operating room for arthroscopic irrigation and debridement, incision and drainage, and subacromial decompression due to right shoulder abscess and right rotator cuff tear. Intraoperatively, findings were remarkable for hemorrhagic synovial and bursal fluid with a small amount of purulent material and full-thickness rotator cuff tear. The orthopedics team continued to follow up while the patient was in house with final recommendations to follow up as an outpatient.

The neurosurgery team was consulted due to the findings of epidural abscesses at T5-T6 and C5-C6 levels with significant canal stenosis. A follow-up MRI with and without contrast was done, with the results showing discitis at the level of T5-T6 with ventral epidural phlegmon. The patient stated at this time that prior to this admission, he was ambulating normally. The physical examination was masked by the presentation of the rhabdomyolysis as described above. Given the full picture of the patient's presentation and in light of the imaging findings, the patient was given the choice to proceed conservatively with aggressive IV antibiotics versus surgical intervention, for which the patient opted in. Consequently, the patient underwent C5 corpectomy, C4 discectomy, C5-C6 discectomy, C4-C6 cage placement, plating of C4-C6, PAS C3-T1, laminectomy C5-C6, fusion, PSF T4-T7, laminectomy T5-T6, decompression and drainage of epidural phlegmon.

As mentioned above, the patient's blood culture was growing *Methicillin-Sensitive Staphylococcus aureus* (MSSA), the patient had a known allergy to penicillin, and he was started on daptomycin and rifampin. Transthoracic echocardiogram followed by transesophageal echocardiogram was done to rule out infective endocarditis.

Blood cultures were persistently positive on five consecutive draws that were 2-3 days apart while the patient was on antibacterial therapy. A negative culture was obtained only after about one month from the first draw; however, the blood culture turned positive shortly after, as the table shows (Table 1).

**Table 1:** Serial blood culture results throughout the hospitalization period. MSSA: Methicillin-sensitive Staphylococcus aureus

Blood culture	Bacteria	Day of hospitalization
Positive	MSSA	1
Positive	MSSA	3
Positive	MSSA	6
Positive	MSSA	7
Positive	MSSA	13
Positive	MSSA	26
Negative	-	30
Positive	MSSA	34
Negative	-	36
Negative	-	51

The infectious disease team was consulted, and a decision was made to proceed with desensitization in the intensive care unit and initiate intravenous nafcillin, given its superior efficacy against *methicillin-sensitive Staphylococcus aureus* (MSSA) and the persistence of positive blood cultures despite alternative antimicrobial therapy (doxycycline, rifampin, and daptomycin).

The patient was subsequently treated with IV nafcillin and oral rifampin for a total duration of six weeks, counted from the date of the first negative blood culture. This was followed by daily oral dicloxacillin for suppressive therapy, to be continued as long as the indwelling hardware remained in place.

On admission, the patient presented with hypokalemia, attributed to gastrointestinal losses secondary to vomiting. This initial electrolyte disturbance resolved promptly with potassium supplementation and cessation of emesis.

However, during the course of IV nafcillin therapy, the patient developed persistent hypokalemia of unclear origin, refractory to daily potassium supplementation. After excluding other potential etiologies, including hyperaldosteronism, diuretic use, gastrointestinal losses, and thyroid dysfunction based on laboratory findings (Table 2) and absence of GI symptoms (vomiting, diarrhea), the hypokalemia was attributed to nafcillin.

**Table 2:** Laboratory work up for hypokalemia

Test	Result	Normal Range
Serum cortisol AM	4.9 ug/dL	6.0 - 18.4 ug/dL
Free T4	1.2 ng/dL	0.9 - 1.8 ng/dL
Free T3	1.35 pg/mL	2.0 - 4.40 pg/mL
Serums Mg	1.7 mg/dL	1.6 - 2.6 mg/dL
Urine Cr	39 mg/dL	30.0 - 150.0 mg/dL
Aldosterone/Renin Ratio	28.6	0.9 - 28.9
Urine potassium	46 mmol/L	25.0 - 125.0 mmol/L
Urine sodium	65 mmol/L	40.0 - 220.0 mmol/L

The calculated fractional excretion of potassium (FEK) (see below for the equation) was about 17.7% which is suggestive of renal potassium wasting.

FEK = (Urine K⁺ × Plasma Creatinine) / (Serum K⁺ × Urine Creatinine) × 100

where FEK represents the fractional excretion of potassium (expressed as a percentage), UK is the urine potassium concentration (in mEq/L), PCr stands for plasma creatinine concentration (in mg/dL), SK denotes the serum potassium concentration (in mEq/L), and UCr refers to the urine creatinine concentration (in mg/dL).

**Figure 4:**
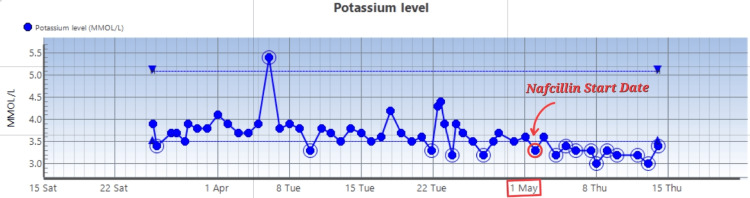
Potassium levels with the yellow highlight showing the date of Nafcillin start

**Figure 5:**
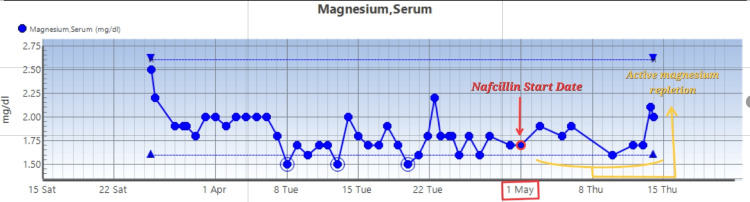
Magnesium levels

US renal artery duplex showed no significant renal artery stenosis. Potassium levels and magnesium levels are as follows (Figures 4, 5).

The core management strategy involved continued electrolyte supplementation during IV nafcillin therapy, consisting of oral potassium chloride 40 mEq twice daily and intravenous magnesium sulfate 1000 mg. Upon discharge, the patient was advised to monitor serum potassium levels closely and maintain daily supplementation with oral potassium chloride 40 mEq and magnesium 400 mg, with a target magnesium level >2 mg/dL to mitigate ongoing potassium losses. The hypokalemia was expected to resolve following completion of the nafcillin course.

The patient was instructed to follow up with his primary care provider to ensure continuity of care. No post-discharge follow-up data were available.

## Discussion

Drug-induced hypokalemia is a common yet frequently underestimated complication in hospitalized patients [4, 5]. Nafcillin, a penicillinase-resistant beta-lactam antibiotic, remains a cornerstone of therapy against MSSA bacteremia which was found to be protective against mortality compared to vancomycin [6]. However, its association with renal potassium wasting is underreported and lacks clear guidelines for monitoring or supplementation.

In this case, the patient developed persistent hypokalemia with a fractional excretion of potassium (FEK) of 17.7%, strongly suggestive of renal potassium loss. The hypokalemia was initially attributed to gastrointestinal losses, but the persistence despite correction and the exclusion of alternative endocrine and renal causes pointed toward nafcillin as the potential culprit.

Nafcillin-associated hypokalemia can be explained by the increased sodium load of the antibiotic preparation on the distal tubule leading to potassium wasting [7], which is the likely mechanism in our case given the increased potassium secretion as discussed above. While there are other suggested mechanisms [4, 8], further studies are required to fully understand them. This pattern of hypokalemia warrants increased clinical vigilance, especially in patients on prolonged courses or with underlying comorbidities that predispose them to electrolyte imbalance.

Identifying nafcillin-induced hypokalemia early enabled timely intervention and prevented further complications such as arrhythmia or muscle weakness. Consistent potassium supplementation and magnesium repletion proved essential for maintaining electrolyte stability during antimicrobial therapy, with discontinuation of the antibiotic remains the definitive solution for this problem.

## Conclusions

This case reinforces the importance of recognizing nafcillin as a potential cause of renal potassium wasting. Clinicians should maintain a high index of suspicion when hypokalemia persists without obvious gastrointestinal or endocrine causes, particularly in the setting of prolonged nafcillin administration. Routine electrolyte monitoring and proactive supplementation should be considered during nafcillin therapy, especially in patients with predisposing factors for hypokalemia. Early recognition and management can minimize morbidity and support safe, uninterrupted antibiotic treatment.
